# Mechanisms of prezygotic post-pollination reproductive barriers in plants

**DOI:** 10.3389/fpls.2023.1230278

**Published:** 2023-07-05

**Authors:** Ludi Wang, Dmitry A. Filatov

**Affiliations:** ^1^ Institute of Biological, Environmental and Rural Sciences (IBERS), Aberystwyth University, Gogerddan, Aberystwyth, United Kingdom; ^2^ Department of Biology, University of Oxford, South Parks Road, Oxford, United Kingdom

**Keywords:** pollen-pistil interactions, molecular mechanisms, prezygotic barriers, self-incompatibility, unilateral incompatibility, plant speciation

## Abstract

Hybridisation between individuals of different species can lead to maladapted or inviable progeny due to genetic incompatibilities between diverging species. On the other hand, mating with close relatives, or self-fertilisation may lead to inbreeding depression. Thus, both too much or too little divergence may lead to problems and the organisms have to carefully choose mating partners to avoid both of these pitfalls. In plants this choice occurs at many stages during reproduction, but pollen-pistil interactions play a particularly important role in avoiding inbreeding and hybridisation with other species. Interestingly, the mechanisms involved in avoidance of selfing and interspecific hybridisation may work via shared molecular pathways, as self-incompatible species tend to be more ‘choosy’ with heterospecific pollen compared to self-compatible ones. This review discusses various prezygotic post-pollination barriers to interspecific hybridisation, with a focus on the mechanisms of pollen-pistil interactions and their role in the maintenance of species integrity.

## 1 Introduction

Although closely related plant species are often cross-compatible and can form hybrids in artificial crosses, the vast majority of species are reproductively isolated in natural populations (Rieseberg et al., 2006). Various interspecific barriers to hybridisation exist at both pre- and postzygotic stages. Prezygotic barriers act before fertilisation, ensuring preferential acceptance of conspecific pollen. Postzygotic barriers act after fertilisation, resulting in hybrid inviability and hybrid breakdown that reduces or prevents reproduction in the next generation. For closely related plant species, prezygotic barriers are believed to play a greater role in reproductive isolation than postzygotic barriers (Rieseberg and Willis, 2007; Widmer et al., 2009; Christie et al., 2022). Different forms of prezygotic isolation in plants can be classified into pre- and post-pollination barriers. Pre-pollination barriers, such as those caused by species-specific pollinators, reduce the possibility of pollen transfer between the plant species. Such barriers have been actively studied; they can be caused by a range of factors, including seasonal reproductive phenology (e.g. flowering at different times) (Gaudinier and Blackman, 2020), ecogeographic adaptation to habitats (Kay, 2006; Schemske, 2010), pollination (Kay and Sargent, 2009; Moreira-Hernández and Muchhala, 2019) and mating system (Markova et al., 2017). On the other hand, post-pollination prezygotic interspecific barriers remain relatively understudied (Broz and Bedinger, 2021; Tran and Lenhard, 2023) and will be the focus of the current review.

Pollen-pistil interaction (PPI) is a series of crucial male-female recognition events that occur after pollination but before fertilisation. PPIs lead to acceptance of intraspecific nonself-pollen and rejection of self-incompatible or interspecific pollen. Reproductive barriers can be established at various stages during PPI, which thereby plays an important role in angiosperm speciation (Bedinger et al., 2017; Hater et al., 2020; Broz and Bedinger, 2021; Hafidh and Honys, 2021). PPI starts with pollen deposition, adhesion and hydration on the stigma, followed by germination and growth of the pollen tube through the style’s transmitting tract to the ovule’s micropyle. After arrival at the micropyle, the pollen tube bursts to release the twin sperm cells to complete fertilisation. These steps involve complex pollen-pistil molecular crosstalk, and ‘miscommunication’, or ‘incongruence’ between interspecific molecules often results in passive incompatibilities, leading to reproductive barriers that reject heterospecific pollen (Heslop-Harrison, 2000; Bedinger et al., 2017; Broz and Bedinger, 2021). In contrast to ‘incongruence’, ‘incompatibility’ involves active mechanisms that affect con- or heterospecific recognition processes (Hogenboom, 1975).

## 2 Self-incompatibility as a barrier to species hybridisation

**Figure 1:**
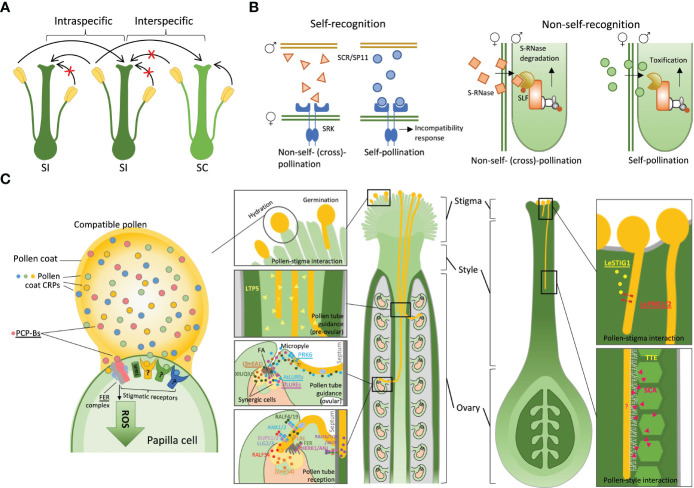
Pollen-pistil interactions during prezygotic post-pollination processes. **(A)** Interspecific and intraspecific interactions between self-incompatible (SI) and self-compatible (SC) species, following the SI x SC rule. **(B)** Self-recognition systems within the Brassicaceae family and non-self-recognition systems within the Solanaceae family. **(C)** Signalling mediated by peptides and receptors during compatible pollen-pistil interactions, including pollen-stigma interactions, pollen tube guidance, and pollen tube reception at the female gametophyte. Question marks highlight unidentified signalling components. Peptides and their receptors interacting in a species-preferential manner are underlined. Abbreviations: SCR, *S*-locus cysteine-rich protein; SRK, *S*-locus receptor kinase; SLF, *S*-locus F-box proteins; PCP-Bs, pollen coat protein B class; FER, FERONIA; SPRI1, Stigma privacy 1; LTP5, lipid transfer protein 5; LeSTIG1, *Lycopersicon esculentum* stigma specific protein 1; LePRK1/2, *Lycopersicon esculentum* pollen-specific receptor kinase 1/2; TTE, transmitting tract epidermis; SCA, stigma/style cysteine-rich adhesin; FA, filiform apparatus; At/TfLURE, *Arabidopsis thaliana* / *Torenia fournieri* LURE; PRK6, pollen-specific receptor-like kinase 6; RALF, rapid alkalization factor; BUPS, budda’s paper seal; ANX, anxur; LRE, LORELEI; ANJ, ANJEA; HERK1, HERCULES RECEPTOR KINASE 1; LLG2/3, (LRE-LIKE GPI-ANCHORED PROTEIN2/3); ZmEA1, *Zea mays* egg apparatus 1; ZmES, *Zea mays* embryo sac.

Self-incompatibility (SI) systems evolved independently in many groups of flowering plants and different angiosperm families have different SI systems that prevent self-fertilisation (Takayama and Isogai, 2005; Allen and Hiscock, 2008). They are usually controlled by a multiallelic *S*-locus, with each SI allele encoding a matching combination of pollen and pistil components of the SI system. The general principle of SI systems is to function as a lock-and-key mechanism triggering pollen rejection or acceptance, but the actual molecular implementations of this principle vary between the plant groups. While the SI systems are ‘designed’ to prevent self-fertilisation, at least some of these systems appear to play a significant role in rejection of heterospecific pollen, creating interspecific reproductive barriers (Kitashiba and Nasrallah, 2014; Broz and Bedinger, 2021; Huang et al., 2023). For a long time, it has been recognised that self-incompatible species have a greater tendency to actively reject heterospecific pollen compared to self-compatible (SC) species (Lewis and Crowe, 1958). The crosses between closely related species often lead to an asymmetric outcome (Tiffin et al., 2001) – unilateral incompatibility (UI), with rejection of heterospecific pollen by SI plants and acceptance of heterospecific pollen by SC plants (Lewis and Crowe, 1958; Li et al., 2018). This pattern, often referred to as the SI × SC rule (**Figure 1A**), suggests that SI is involved in heterospecific pollen rejection.

Based on the current understanding of the SI mechanisms, they can be classified into self- and non-self-recognition systems (**Figure 1B**) (Fujii et al., 2016). It may appear improbable that the self-recognition SI, such as those found in Brassicaceae (Murase et al., 2020) and Papaveraceae (Goring et al., 2023), can contribute to interspecific reproductive isolation. In these plant families, the SI reaction causing pollen rejection has to be switched on by the right combination of pollen and pistil components (Kachroo et al., 2001; Takayama et al., 2001) (**Figure 1B**). Genes encoding the matching ‘lock’ and ‘key’ components (*S* proteins) are always kept together – linked in the same allele (haplotype) of the *S*-locus, where recombination is suppressed (Takayama and Isogai, 2005). Thus, recognition triggering pollen rejection occurs only for the pollen and pistil proteins encoded by the same haplotype in the *S*-locus, which is very specific and effective to prevent self-fertilisation, but may not seem particularly suited for rejection of heterospecific pollen that would not bear the right ‘key’ to trigger the SI. Nevertheless, the recent findings in Brassicaceae indicate that self-recognition SI systems can also be involved in heterospecific pollen rejection (Takada et al., 2017; Huang et al., 2023). Takada et al. (2017) identified a novel pair of *S* proteins, a stigma receptor SUI1 (Stigma unilateral incompatibility 1) and a pollen ligand PUI1 (Pollen unilateral incompatibility 1), which are similar to the *S* proteins in *Brassica* SI, *S*-locus receptor kinase (SRK) and *S*-locus cysteine-rich protein (SCR) (Schopfer et al., 1999; Takasaki et al., 2000) (**Figure 1B**). The interaction of SUI1 and PUI1 governs unilateral incompatibility between distinct populations, suggesting a potential molecular mechanism whereby SI signalling contributes to reproductive barriers in allopatry. The recent study by Huang et al. (2023) revealed that the *Brassica* SI female determinant, SRK, not only rejects self-pollen but also rejects interspecific pollen, demonstrating the capacity of self-recognition SI systems in establishing reproductive barriers.

The non-self-recognition SI systems (**Figure 1B**), such as those found in Solanaceae, Plantaginaceae, Rosaceae and Rutaceae (Takayama and Isogai, 2005; Fujii et al., 2016; Liang et al., 2020) appear more readily suited to prevent both self-fertilisation and interspecific hybridisation. In such SI systems, the barrier to pollen growth is ‘always on’ and by default rejects all pollen except the pollen that has the right resistance components. Pollen rejection is caused by S-RNase that is encoded by the *S*-locus and is expressed in the pistil (McClure et al., 2011). Cytotoxic effects caused by S-RNase inhibit pollen tube growth. Pollen resistance to S-RNase is conferred by the *S*-locus F-box proteins (SLFs) that are expressed in pollen and mediate S-RNase ubiquitination and degradation (Sun et al., 2018). S-RNase gene is highly polymorphic, with multiple alleles present in the same species (Liang et al., 2020). For the SI system to function, a pollen grain has to have resistance to most S-RNase alleles present in that population, except the allele in the same *S*-haplotype. This is achieved by the presence of multiple SLF genes in each *S*-haplotype, which collectively recognise all non-self S-RNases (Fujii et al., 2016). Thus, RNase-based SI is often referred to as a collaborative non-self recognition system (Kubo et al., 2010).

Because by default S-RNase prevents grown of any non-resistant pollen, it is easy to see how it can cause rejection of heterospecific pollen and prevent hybridisation with other species. However, this system may not provide an effective barrier from the pollen of closely related species that have the same SI and share the SLF resistance genes in the *S*-locus. The involvement of S-RNase in rejection of heterospecific pollen was tested in tobacco (Murfett et al., 1996) and tomatoes (Covey et al., 2010; Qin et al., 2018), where both S-RNase dependent and independent rejection (Tovar-Mendez et al., 2017) of heterospecific pollen was observed, illustrating the complexity of heterospecific pollen rejection, with multiple redundant mechanisms involved. The pollen components of SI in RNase-based SI – the F-box proteins were also revealed to be involved in pollen rejection in unilateral incompatibility (Li and Chetelat, 2015), which supports the role of SI in heterospecific pollen rejection.

## 3 Stages of pollen-pistil interactions

Below we discuss different stages of pollen-pistil interactions when SI-dependent or independent rejection of heterospecific pollen occurs, leading to reproductive barriers.

### 3.1 Pollen-stigma interactions

After pollination, the first stage of the pollen-pistil interactions occurs right at the surface of the stigma (**Figure 1C**). The stigma is the receptive terminal of the pistil (Bedinger et al., 2017) and can be classified into two broad groups based on their structure and size: wet stigmas and dry stigmas (Heslop-Harrison and Shivanna, 1977). Wet stigmas, such as in *Nicotiana* and *Petunia* (Solanaceae), are covered by a viscous secretion, while dry stigmas, e.g. in Brassicaceae (Edlund et al., 2004), lack a surface exudate and possess a layer of intact papilla cells. There are also semi-dry stigmas, such as those found in the Asteraceae family, where a small amount of exudate is present when the stigma is mature (Hiscock et al., 2002).

Once the pollen grain is deposited on the stigma surface, it must be hydrated to become metabolically activated before germinating and developing a pollen tube that grows through the pistil to deliver the sperm cells to the ovary for fertilisation. Wet stigmas tend to hydrate pollen indiscriminately, while pollen hydration on dry stigmas tends to be more selective (Heslop-Harrison, 2000). In Brassicaceae, the dry stigma surface is a highly discriminative first point of contact with the desiccated pollen grains (Huang et al., 2023), which are reliant on the stigma papilla cells to provide water and other components required for pollen hydration and germination (Broz and Bedinger, 2021). The evolution of the dry stigma made pollen hydration a crucial checkpoint in the reproductive process. At this stage, a molecular dialogue occurs between the pollen grain and the stigmatic papilla cell to determine their compatibility. Therefore, it is at this stage where the earliest post-pollination prezygotic barrier can be established, contributing to the prevention of fertilisation between different plant species.

The pollen coat, which is the outermost layer of the pollen grain, plays a central role in mediating molecular recognition events during pollen-stigma interactions. Accumulating evidence has demonstrated that the pollen coat carries small secreted cysteine-rich proteins (CRPs) that are involved in cell-cell communication during pollen–stigma interactions (**Figure 1C**) (Bircheneder and Dresselhaus, 2016; Kim et al., 2021; Wang et al., 2023). Despite being diverse and mostly functionally uncharacterised, CRPs have been identified as key players in plant reproductive signalling (Silverstein et al., 2007; Marshall et al., 2011; Kim et al., 2021). Genes encoding these signalling proteins are often highly polymorphic and species- or genus-specific, suggesting their important roles in establishing and maintaining reproductive barriers between species. Among these CRPs are the pollen coat protein B class (PCP-Bs), which regulate compatible pollen hydration (Wang et al., 2017) by competing with stigmatic ligands for binding to the stigmatic receptor FERONIA (FER) complex, leading to a reduction in stigmatic reactive oxygen species (ROS) that facilitates pollen hydration (**Figure 1C**) (Liu et al., 2021; Huang et al., 2023). PCP-B are polymorphic among species in Brassicaceae, and their binding with FER occurs in a species-preferential manner. *Arabidopsis* PCP-Bγ outcompetes PCP-B from *Brassica rapa* for binding to FER, thereby serving as a ligand-receptor pair in establishing an interspecific reproductive barrier during pollen-stigma interaction (Huang et al., 2023).

A recently identified *Arabidopsis* stigma-specific transmembrane protein, Stigma privacy 1 (SPRI1), confers interspecies incompatibility by rejecting pollen from distantly related species in Brassicaceae (Fujii et al., 2019). This finding proposes a novel SI-independent mechanism for promoting intraspecific pollen germination, that in turn, facilitates reproductive isolation. However, the pollen ligands that interact with SPRI1 are yet to be identified. Recent proteomic studies of the pollen coat of three Brassicaceae species revealed numerous CRPs with uncharacterised functions (Wang et al., 2023), which provides possible candidates for mediating both intra- and interspecific pollen-stigma recognition by interacting with SPRI1 and other unknown stigmatic receptors (**Figure 1C**). Some of the genes encoding these pollen coat CRPs, including PCP-Bs, are undergoing rapid diversification and evolving under positive selection (Wang et al., 2023). Evolutionary analysis revealed that SPRI1 function was lost multiple times during the evolution of *A. thaliana* (Fujii et al., 2019). Impaired hydration or germination of pollen due to lower ligand-receptor-binding affinity can lead to a delay in pollen tube growth. Thus alternations in either ligands or receptors apply selective pressure on genes encoding the binding partners, suggesting that the coevolution between PCP-B and FER, as well as between SPRI1 and its as-yet-unidentified pollen ligand, may play a more significant role in shaping the species-specificity of their interaction, rather than the evolution of the ligands alone. Consequently, this coevolution could contribute substantially to the establishment of prezygotic barriers and the process of speciation.

In tomato (*Solanum lycopersicum*), a small pistil secreted CRP, STIGMA-SPECIFIC PROTEIN1 (STIG1), was identified as a signalling ligand that binds to the pollen receptor kinase (LePRK1/2) (**Figure 1C**). This interaction promotes pollen tube growth by regulating cellular reactive oxygen species (ROS) production (Goldman et al., 1994; Verhoeven et al., 2005; Huang et al., 2014). Although it is not clear whether STIG1 and LePRK1/2 interact in a species or family-preferential manner, studies suggested that STIG1 homologues have diverged functions in Solanaceae species (Goldman et al., 1994; Verhoeven et al., 2005; Huang et al., 2014), indicating the potential role of STIG1 in establishing a prezygotic reproductive barrier during early pollen tube growth.

### 3.2 Pollen-style interactions

Pollen tube growth through the style towards the ovary is another important phase contributing to interspecific reproductive barriers. The styles vary widely in size and structure, making the speed and navigation of pollen tube growth through the style crucial for reproductive success. Multiple (mostly not yet identified) molecular factors involved in pollen tube-style interactions appear to contribute to interspecific hybridisation barriers.

In *Solanum*, silencing the genes encoding pistil SI proteins, HT proteins, eliminates the stylar barrier in *S. lycopersicum* to heterospecific pollen from *S. habrochaites* and *S. arcanum.* However, this silencing only weakens but does not eliminate the barrier for *S. pennellii* pollen, implying the presence of additional female barriers (Tovar-Mendez et al., 2017). S-RNase-based self-incompatibility in Solanaceae is known to play an important role in the prevention of interspecific hybridisation (Chetelat and Deverna, 1991; Murfett et al., 1996; Baek et al., 2015; Bedinger et al., 2017). On the pollen side, multiple genetically identified loci confer pollen resistance to S-RNase-based hybridisation barriers between *S. pennellii* and *S. lycopersicum* (Chetelat and Deverna, 1991). Some of these genes have been identified and functionally characterised. In particular, *SpSLF-23* gene linked to the *S*-locus and *Cullin1* gene encoding CUL1 that is part of Skp1-Cullin-F-box ubiquitin E3 ligase complex in *S. pennellii* target pistil SI factor for degradation to unilaterally overcome the interspecific barrier (Chetelat and Deverna, 1991; Li and Chetelat, 2010; Li and Chetelat, 2014; Li and Chetelat, 2015). Overexpression of a gene encoding farnesyl pyrophosphate synthase in the pollen of *S. lycopersicum* has been shown to cause resistance to S-RNase-independent interspecific incompatibility (Qin et al., 2018).

Some plants, such as lilies, have open styles and the pollen tube grows through mucilage covering the surface of the central canal. In *Lilium longiflorum*, stigma/style cysteine-rich adhesin (SCA) peptides, which are members of lipid transfer proteins (LTPs) belonging to CRPs, play a crucial role in the adhesion of pollen tubes to the stylar transmitting tract epidermis (TTE) (**Figure 1C**) (Park et al., 2000; Chae et al., 2007). The TTE secrets SCA peptides, which facilitate the adhesion of pollen tubes to the TTE wall surface by forming an adhesive matrix with the pollen tube through binding to stylar pectin in a pH-dependent manner (Mollet et al., 2000). In *A. thaliana*, an SCA-like LTP5 is secreted from both pollen and the pistil and is essential for maintaining normal pollen tube growth and fertility (Chae et al., 2009) (**Figure 1C**). Although no protein receptor has been identified for the SCA/pectin complex, interspecific differences in the density of extracellular matrix and functions of enzymes produced by the pollen tube suggest that this pollen tube-style adhesion regulatory system may provide a platform for the establishment of reproductive barriers by promoting or opposing pollen tube growth, favouring conspecific pollen.

### 3.3 Pollen-ovary interactions

The interaction between the growing pollen tube and the ovary marks the final stage at which post-pollination prezygotic interspecific barriers can act. Once a pollen tube grows through the transmitting tract (TT) and emerges onto the septum surface, it must be precisely guided towards the micropyle for sperm cell delivery. Studies in various species have shown that attraction of pollen tubes to the embryo sac and ovules can occur in a species-specific manner (Higashiyama et al., 2006; Uebler et al., 2013; Zhong et al., 2019), which may play a crucial role in establishing reproductive barriers between plant species. Several molecular factors involved in pollen-ovary interactions appear to contribute to interspecific hybridisation barriers. In *Zea mays*, a small peptide, EGG APPARATUS1 (ZmEA1), is expressed specifically in the synergid cells and filiform apparatus (FA), and it attracts pollen tubes while arresting their growth at higher concentrations (**Figure 1C**) (Márton et al., 2005; Márton et al., 2012). Moreover, it binds to the pollen tube in a species-preferential manner. When expressed in *Arabidopsis* ovules, ZmEA1 guides maize pollen tubes to grow towards the micropylar opening of the ovule *in vitro* (Márton et al., 2012). A group of synergid-expressed defensin-like CRPs identified in *Torenia* and *Arabidopsis*, LURE (TfLUREs and AtLUREs), *in vitro* demonstrated activity in attracting pollen tube growth in a species-specific manner (Higashiyama et al., 2006; Okuda et al., 2009; Takeuchi and Higashiyama, 2012). Pollen tube tip-localised receptor-like kinase 6 (PRK6) acts as an essential receptor for perceiving the signalling of the LURE1 peptides in *A. thaliana* (AtLURE1), guiding the pollen tube towards the ovule (Takeuchi and Higashiyama, 2016). A recent study has identified four LURE1-related peptides, XIUQIU1-4, as pollen tube attractants that act irrespective of the species and independently of PRK6 in Brassicaceae (Zhong et al., 2019) (**Figure 1C**). The AtLURE1s-PRK6 interactions were revealed to be not essential that fertilisation but rather facilitate the emergence of pollen tubes onto the septum surface, promoting the selection of conspecific pollen (Zhong et al., 2019). Phylogenetic profiling demonstrated that each XIUQIU peptide has ortholog(s) in the analysed species of Brassicaceae, while AtLURE1s form species-specific clusters within *Arabidopsis* species, reflecting their distinct functions in species-specificity and roles in reproductive isolation (Takeuchi and Higashiyama, 2012; Zhong et al., 2019).

The species-specificity of molecular crosstalk during pollen tube reception may also contribute to interspecific reproductive barriers. Incongruity of molecular mechanisms at this stage can result in pollen tube overgrowth caused by failure of growth arrest and tip rupture to release sperm cells. This phenotype has been observed in interspecific crosses in *Arabidopsis* (Escobar-Restrepo et al., 2007) and *Rhododendron* (Williams et al., 1986). In maize, pollen tube rupture upon arrival at the micropyle is mediated by cysteine-rich defensin-like proteins ZmES4 (Amien et al., 2010), which are released from vesicles in the egg apparatus (**Figure 1C**). In *Arabidopsis*, the regulation of pollen tube rupture is different, and it involves a group of signalling peptides called Rapid Alkalinisation Factors (RALFs), which also belong to the CRPs. RALFs and members of *Catharanthus roseus* RLK1-LIKE (CrRLK1L) receptors regulate pollen tube reception, tip rupture and the prevention of polytubey (Ge et al., 2017; Mecchia et al., 2017; Galindo-Trigo et al., 2020; Zhong et al., 2022) (**Figure 1C**). As the pollen tube reaches the micropyle, a synergid receptor complex comprising FER, LORELEI (LRE), ANJEA (ANJ) and HERCULES RECEPTOR KINASE 1 (HERK1) controls pollen tube reception through perceiving unidentified pollen ligands (Galindo-Trigo et al., 2020). Autocrine pollen ligands RALF4/19 interact with the pollen tube receptor complex anxur/budda’s paper seal (ANX/BUPS), maintaining cell wall integrity and preventing premature rupture. Synergid-secreted RALF34 competes with RALF4/19 for binding to the ANX/BUPS-LLG2/3 (LRE-LIKE GPI-ANCHORED PROTEIN2/3) complex, triggering the rupture of the pollen tube and the release of sperm cells (Ge et al., 2017; Ge et al., 2019). Septum-localised FER-ANJ-HERK1 receptor complex interacts with pollen ligands RALF6, 7, 16, 36 and 37 to regulate polytubey blocking (Zhong et al., 2022) (**Figure 1C**). While this mechanism appears to differ between distantly related species, it is not clear whether it contributes significantly to interspecific reproductive barriers between closely related species.

## 4 Conclusion and perspective

Reproductive barriers play a critical role in maintaining the boundaries between plant species. Pollen-pistil interaction, including the recognition of self-incompatible or heterospecific pollen, is a key step in prezygotic isolation, but the molecular mechanisms of the reproductive barriers established through PPI are not yet fully understood. Self-incompatibility (SI) systems contribute to reproductive isolation between plant species, with non-self-recognition SI systems likely playing a particularly important role in interspecific prezygotic reproductive isolation, but the role of self-recognition SI in heterospecific pollen rejection is also starting to emerge (Huang et al., 2023). Although significant progress has been made in identifying molecular factors that contribute to prezygotic post-pollination reproductive barriers, many more remain to be discovered. Research on PPI-based mechanisms of reproductive isolation has been limited to very few (mostly model) species. Extending this investigation to a wider range of non-model organisms and uncovering additional signalling proteins and their binding partners involved in cell-cell recognition, along with conducting phylogenetic studies on these factors, will enhance our ability to make informative comparisons of PPI evolution and to unravel their significance in reproductive isolation and speciation.

While prezygotic post-pollination reproductive barriers are important for preserving species integrity and preventing interspecific hybridisation, it remains unclear whether they play a significant role in the formation of new species. The discovery of a pollen-stigma barrier between different *Brassica* populations caused by a duplication of *S*-locus (Takada et al., 2017) suggests that PPIs may be a significant driver of speciation in flowering plants. However, more species need to be analysed to identify additional cases where PPIs have been or are driving speciation and to evaluate their significance in plant speciation.

## Author contributions

LW and DAF conceived this review and wrote the manuscript.
